# Use of large pieces of printed circuit boards for bioleaching to avoid ‘precipitate contamination problem’ and to simplify overall metal recovery

**DOI:** 10.1016/j.mex.2014.08.011

**Published:** 2014-08-30

**Authors:** N.N. Adhapure, P.K. Dhakephalkar, A.P. Dhakephalkar, V.R. Tembhurkar, A.V. Rajgure, A.M. Deshmukh

**Affiliations:** a Department of Microbiology, Dr. B.A.M. University, Sub-campus, Osmanabad 413501, M.S., India; b Microbial Sciences Division, Agharkar Research Institute, Pune, M.S., India; c N.K.S.P.T's Arts, Science and Commerce College, Badnapur, Dist. Jalna 431202, M.S., India; d School of Physical Science, Solapur University, Solapur 413001, M.S., India

**Keywords:** Microbial consortium, Large pieces of printed circuit boards, Bioleaching

## Abstract

Very recently bioleaching has been used for removing metals from electronic waste. Most of the research has been targeted to using pulverized PCBs for bioleaching where precipitate formed during bioleaching contaminates the pulverized PCB sample and making the overall metal recovery process more complicated. In addition to that, such mixing of pulverized sample with precipitate also creates problems for the final separation of non metallic fraction of PCB sample. In the present investigation we attempted the use of large pieces of printed circuit boards instead of pulverized sample for removal of metals. Use of large pieces of PCBs for bioleaching was restricted due to the chemical coating present on PCBs, the problem has been solved by chemical treatment of PCBs prior to bioleaching. In short,

• Large pieces of PCB can be used for bioleaching instead of pulverized PCB sample.
• Metallic portion on PCBs can be made accessible to bacteria with prior chemical treatment of PCBs.
• Complete metal removal obtained on PCB pieces of size 4 cm × 2.5 cm with the exception of solder traces. The final metal free PCBs (non metallic) can be easily recycled and in this way the overall recycling process (metallic and non metallic part) of PCBs becomes simple.

## Graphical abstract

**Figure:**
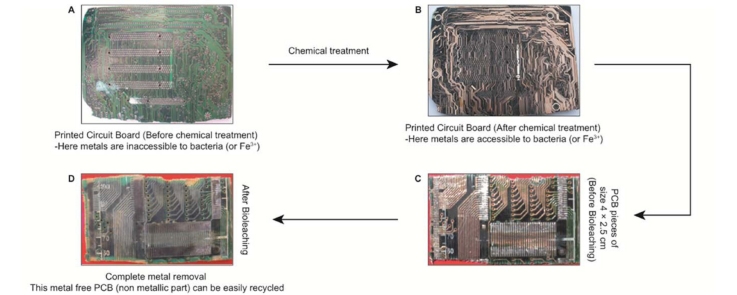

## Method details

There were some attempts of using bioleaching for removal of metals from electronic waste [1–5,7,8]. Most of the research workers have used powdered sample/pulverized sample of waste printed circuit board for removal of metals [1,3,5,7,8].

## ‘Precipitate contamination’ problem

Normally precipitate formation is common in any bioleaching process. The precipitate is generally composed of ferric hydroxides. In the experiments of bioleaching of metals from pulverized PCBs, the precipitate formed during the process is composed of Sn, Cu, Pb and Fe [4,5]. Occurrence of such precipitate makes it difficult to distinguish between the precipitate and residual PCB powder [1]. It means the formed precipitate is contaminating the PCBs (pulverized) sample and making the overall metal recovery process more complicated. In addition to that, such mixing of pulverized sample with precipitate also creates problems for the final separation of non metallic fraction of PCB sample.

However, if large pieces of printed circuit boards were used for metal removal and complete metal removal is achieved, then the remaining board (non metallic part) could be easily recycled; which is otherwise difficult while using pulverized PCBs. Hence large pieces of PCBs should be preferred over pulverized PCBs so as to simplify the overall recycling (metallic as well as non metallic fraction) process and also to avoid the problem of ‘precipitate contamination’. The main problem of using large pieces of PCBs for bioleaching is the chemical coating present on the PCBs which does not allow the bacteria (or Fe^3+^) to penetrate through it and thus the bacteria (or Fe^3+^) fail to reach the metal. Removal of chemical coating prior to bioleaching can solve the problem.

In the present investigation we attempted the use of large pieces of printed circuit boards instead of pulverized sample for removal of metals.

The method includes following steps:

1. Collection of PCBA, physical removal of plastic parts viz. RAM, PCI slot, chip slots from PCBA.
2. Chemical treatment of PCB.
3. PCBs were cut in different sizes (either 12 cm × 6 cm or 4 cm × 2.5 cm).
4. Bioleaching process.
5. Analysis of metals in leachate by AAS, analysis of Fe^2+^ and pH of leachate.

## Collection of PCBA

Printed circuit board assemblies (PCBA) were collected from scrap market. Attached plastic parts viz. RAM, PCI slot, chip slots were removed from printed circuit board (PCB). These PCBs were used for chemical treatment as mentioned below.

## Chemical treatment of PCBs

**Fig. 1:**
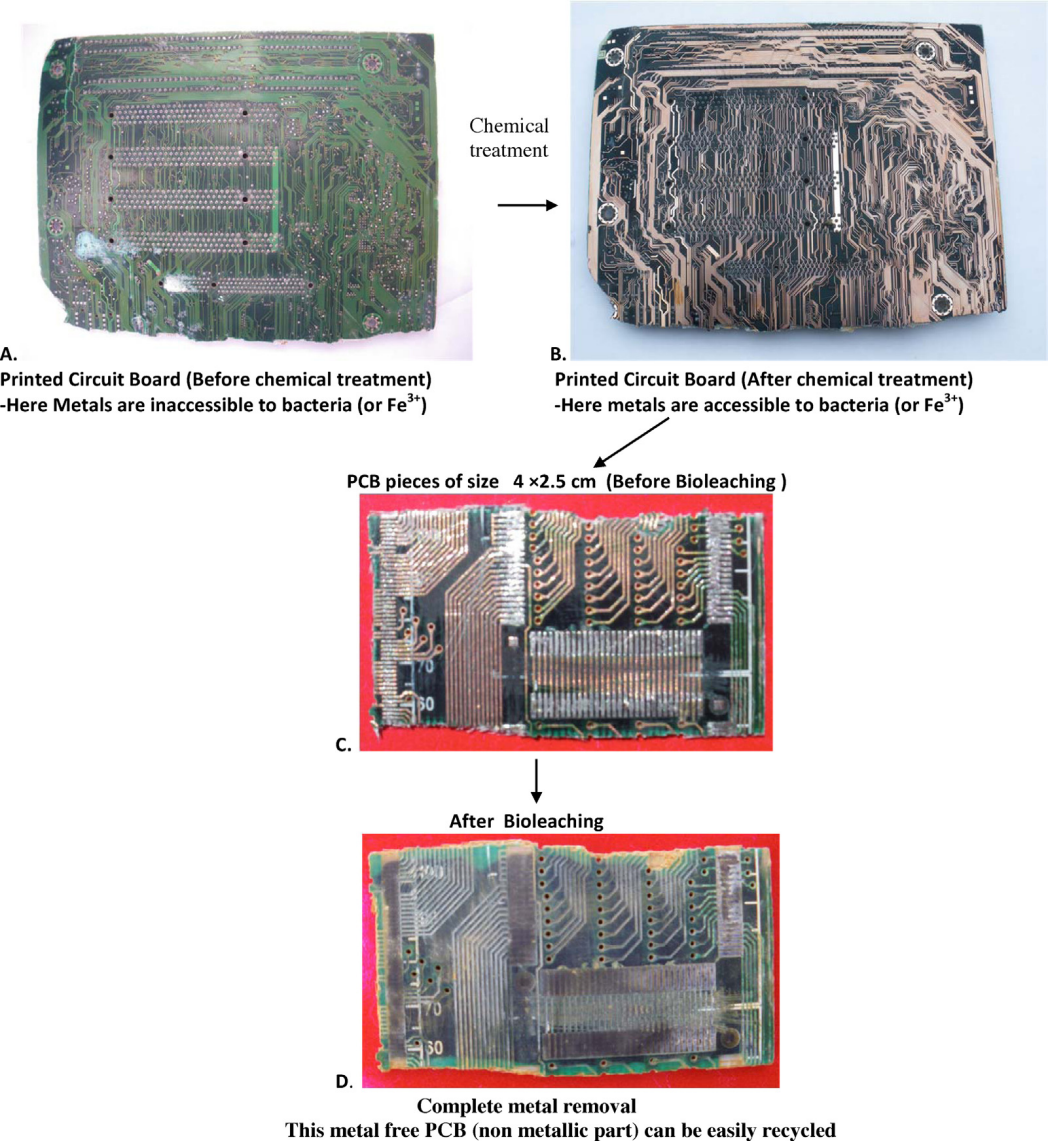
Simplified process of bioleaching of metals from waste printed circuit boards.

The PCBs (Fig. 1A) if directly placed in contact with MMC (Mixed Microbial Consortium), it results in no leaching of metals. This was due to the chemical coating (solder mask) on the PCBs. The commonly used material for solder mask is epoxy. Solder mask does not allow the bacteria (or Fe^3+^) to penetrate through it and thus the bacteria (or Fe^+3^) fail to reach the metal. Removal of such chemical coating (solder mask) can fascilitate use of large pieces of PCBs for bioleaching. Considering this, several chemicals were tried to remove the coating and it was observed that 10 M NaOH gives better results. So, PCBs were dipped overnight in a 10 M NaOH and then washed under running tap water. The washed water was replaced by fresh distilled water until the adhered NaOH was removed (approximately 4–5 times). This was monitored by determining pH of washed water. Neutral pH of washed water confirms the complete removal of NaOH. The washed PCBs (Fig. 1B) were then used further.

## PCBs were cut in different Sizes

The washed PCBs (Fig. 1B) were then cut in different sizes (12 cm × 6 cm and 4 cm × 2.5 cm) and separately used for bioleaching.

## Bioleaching process

**Fig. 2:**
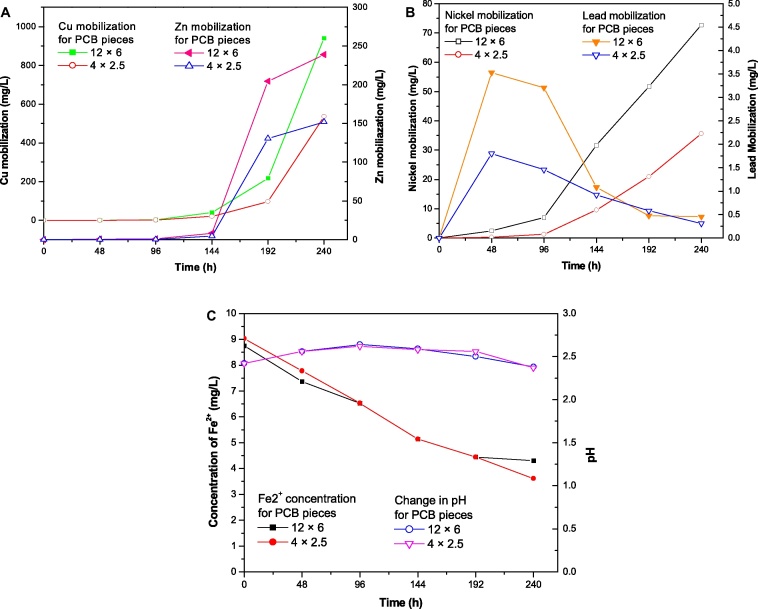
(A and B) Bioleaching of metals (Cu, Zn, Ni and Pb) from large pieces of PCB having size 12 cm × 6 cm and 4 cm × 2.5 cm. (C) Changes in concentration of Fe^2+^ and pH during bioleaching.

After chemical treatment (Fig. 1) and subsequent washing the printed circuit boards were cut in respective sizes (12 cm **×** 6 cm and 4 cm **×** 2.5 cm) and were used for bioleaching. PCB pieces (size 12 cm **×** 6 cm) were subjected to bioleaching by MMC and metal concentration in leachate was determined by AAS (Fig. 2A and B).

### Microorganisms used

Microorganisms to be used for the experiments were obtained from natural environment. Two ore samples namely bauxite and pyrite were used for enrichment of acidophiles. The enrichment was carried out in modified 9K medium having composition (g/l): part A—(NH_4_)_2_SO_4_ – 3.0; KCl – 0.20; K_2_HPO_4_ – 0.050; K_2_HPO_4_ – 0.050; MgSO_4_**·**7H_2_O – 0.050; part B—FeSO_4_**·**7H_2_O – 45. Part A was sterilized by autoclaving at 121 °C for 15 min at 15 lb pressure and part B was sterilized by filtration using 0.25 μm filter (Merck Millipore, United States). After sterilization equal amount of part B was added to part A, aseptically. The pH of the medium was adjusted to 2.4 using 1.0 N H_2_SO_4_. The enriched acidophiles from both the flasks were then mixed. This mixed culture of microorganisms was named as MMC (Mixed Microbial Consortium) and was again cultivated in modified 9K medium. This MMC was used throughout the bioleaching experiment keeping in mind the top down approach [6]. The experiments were conducted in non-sterile conditions.

### Bioleaching of printed circuit board pieces (size 12 cm × 6 cm)

PCB piece having size 12 cm × 6 cm was added in an individual plastic tray. 1 l of modified +9K medium was added in each tray. pH was adjusted to 2.42. 15% MMC inoculum was added and the tray was kept on orbital rotating shaker adjusted at 50 rpm at 30 °C for 10 days. Samples were taken for every 48 h up to 10 days for determination of pH, ferrous iron content and soluble metal content. pH was measured using a digital pH meter, ferrous iron content was measured by n-phenylanthranilic acid method. Soluble metal content (Cu, Pb, Zn and Ni) was detected using atomic absorption spectrophotometer (Perkin Elmer A Analyst 300).

### Bioleaching of printed circuit board pieces (size 4 cm × 2.5 cm)

Printed circuit boards were cut into pieces of having average size 4 cm × 2.5 cm (Fig. 1C). Two pieces were added in the 500 ml capacity Erlenmeyer flasks containing 200 ml modified +9K medium. pH was adjusted to 2.42. MMC inoculum (15%) was added in it and the flasks were kept for incubation on orbital rotary shaker adjusted to 120 rpm at 30 °C. Samples were taken for every 48 h up to 10 days for determination of pH, ferrous iron content and soluble metal content. pH was measured using a digital pH meter, ferrous iron content was measured by n-phenylanthranilic acid method and soluble metal content (Cu, Pb, Zn and Ni) was detected using atomic absorption spectrophotometer (Perkin Elmer A Analyst 300).

It was found that, 940 mg l^−1^, 239 mg l^−1^ and 72.7 mg l^−1^ of Cu, Zn and Ni respectively were solubilized in 240 h for PCB pieces of size 12 cm **×** 6 cm. However in case of Pb it was found that, maximum solubilization achieved was 3.53 mg l^−1^ after 48 h.

In case of Cu, Zn and Ni the metal solubilization was found to increase with time. However maximum Cu leaching during 192–240 h and maximum Zn leaching during 144–192 h was observed.

The pH of the medium was measured after every 48 h and it was found that the pH was changes from 2.42 to 2.38 during incubation of 10 days (Fig. 2C). The possible reasons for increase in pH are alkalinity of PCB powder, consumption of protons during biological oxidation of ferrous iron and consumption of protons by metals or metal oxides.

Ferrous iron content of the medium was determined after every 48 h. Ferrous oxidation pattern can be observed from Fig. 2C. At the end of 10th day the ferrous iron content was 4.305 g/l.

PCB pieces (size 4 cm **×** 2.5 cm) were subjected to bioleaching by MMC in a conical flask and metal concentration in leachate was determined by AAS (Fig. 2A and B). It was found that 536 mg l^−1^, 152 mg l^−1^, 35.64 mg l^−1^ and 0.31 mg l^−1^ of Cu, Zn, Ni and Pb was solubilized in 240 h of incubation. However in case of Pb it was found that, maximum solubilization achieved was 1.8 mg l^−1^ after 48 h.

The pH of the medium was observed after every 48 h during incubation of 240 h (Fig. 2C). It was found that the pH increases in initial phase and decreases later. The ferrous iron content of the medium was measured during incubation after every 48 h and mentioned in Fig. 2C. It was noted that the ferrous iron content decreases slowly as compared to normal iron oxidation by MMC.

After 10 days of incubation the pieces of circuit board were observed, it was found that the metals which were there on circuit board were completely mobilized, in liquid medium. Better clearance of PCB pieces was observed for 4 cm × 2.5 cm as compared to 12 cm **×** 6 cm.

It was observed that complete metal removal (Fig. 1D) takes place in 10 days from the PCB pieces of size 4 cm × 2.5 cm. However the PCB pieces bearing solder part showed traces of solder left on PCB pieces even after bioleaching. This shows poor lead solubilizing activity of MMC.

The use of PCB pieces instead of using PCB powder for bioleaching is quite simple, easier and also saves a cost of powder preparation. After complete removal of metals from PCB pieces, these metal free PCB pieces can be directly used for further recycling. According to authors knowledge this was a first attempt of using large pieces of printed circuit boards for bioleaching. There was a problem for use of large pieces of PCBs. The dye which is present on PCBs prevents access of bacteria to the metals. The dye may be green, blue, orange or red in color. The dye was removed by 10 M NaOH. In this way the metals on the PCBs were made accessible to the bacteria.

As large pieces of PCBs were used for metal removal and complete metal removal was achieved, the remaining board (Fig. 1D) (non metallic part) could be easily recycled; which is otherwise difficult while using pulverized PCBs. Hence large pieces of PCBs should be preferred over pulverized PCBs so as to simplify the overall recycling (metallic as well as non metallic fraction) process and also to avoid the problem of ‘precipitate contamination’.

The experiments were conducted in non-sterile conditions by keeping in mind the conditions of commercial bioleaching plant [6].

The developed microbial consortium at the end of experiment was used for identification of acidophiles present in it. By using PCR amplicons of 16S rDNA fragments with species specific primers, it was confirmed that there is a presence of *Acidiphilum* spp., *Leptospirillum* spp., *Thiobacillus ferroxidans*, *Thiobacillus caldus* and *Sulfobacillus* in the MMC.

Thus the present study concludes that large pieces of PCBs should be used for bioleaching instead of pulverized PCBs. Use of large pieces of PCB is facilitated by chemical pretreatment of PCBs before bioleaching. Complete metal removal was observed on PCB pieces having size 4 cm × 2.5 cm with the exception of solder traces. As the non sterile conditions are used throughout the experiment the consortium would be competitive in conditions of commercial bioleaching plant.
